# Association between bone mineral density and stroke: a meta-analysis

**DOI:** 10.3389/fneur.2025.1561559

**Published:** 2025-03-26

**Authors:** Peng Zhao, Huaxia Sun

**Affiliations:** ^1^Department of Neurology, The Second Affiliated Hospital of Hebei North University, Zhangjiakou, Hebei Province, China; ^2^Department of Neurology, Weifang Hospital of Traditional Chinese Medicine, Weifang, Shandong Province, China

**Keywords:** bone density, osteoporosis, osteopenia, stroke, cerebrovascular accident

## Abstract

**Objective:**

The correlation between bone mineral density (BMD) and stroke remains inconsistent. This study aims to determine whether a reduction in BMD is associated with an increased risk of stroke.

**Methods:**

We systematically searched Medline, Embase, and the Cochrane Database of Systematic Reviews through January 2025 to identify cohort studies with follow-up that reported the influence of a reduction in BMD or low BMD status on the risk of any type of stroke. Pooled analyses were performed using random-effects models.

**Results:**

This study included 13 studies with 146,758 individuals. A 1 SD reduction in BMD was associated with an increased risk of stroke (eight studies; RR, 1.24; 95% CI, 1.09–1.40; *p* < 0.001; I^2^ = 87%). Subgroup analysis showed that a per SD reduction in BMD was associated with incident stroke in female persons (eight studies; RR, 1.28; 95% CI, 1.09–1.51; *p* = 0.002; I^2^ = 87%), but not in male persons (four studies; RR, 1.04; 95% CI, 0.99–1.10; *p* = 0.15; I^2^ = 0%). People with osteoporosis or osteopenia had an increased risk of incident stroke (six studies; RR, 1.59; 95% CI, 1.22–2.08; *p* < 0.001; I^2^ = 92%), as well as male persons (two studies; RR, 3.16; 95% CI, 1.96–5.12; *p* < 0.001; I^2^ = 35%). Sensitivity analysis showed that the results were stable.

**Conclusion:**

Reduction in BMD is associated with a significantly increased risk of stroke. Female individuals have a higher risk than male persons.

## Introduction

Stroke is the second leading cause of mortality and a leading cause of long-term disability across the world (1, 2). The financial burden associated with stroke treatment is enormous. There are approximately 4 million new stroke cases in China and 0.7 million in the United States every year (3, 4). Identifying the underlying risk factors is essential for implementing effective prevention strategies to decrease the risk of stroke and mitigate the disease burden.

Low bone mineral density (BMD) is a significant public health problem, especially among women. It has been well-recognized that individuals with stroke have an increased risk of low BMD or hip fractures (5, 6). However, the role of BMD in stroke risk remains controversial. Myint et al. (7) reported that low BMD was a risk factor for subsequent stroke in the middle and older age healthy general population. In the same research, the meta-analysis of 4 studies found an inverse relationship between BMD and the risk of incident stroke (7). A recent study with a large sample size found no evidence for the association between a decrease in BMD and incident stroke (8). Another meta-analysis reported that BMD was not associated with the risk of stroke mortality (9). Current conclusions are not consistent.

Hence, we conducted a systematic review and meta-analysis in the present study to synthesize available evidence. The primary objective was to determine whether low BMD is associated with an increased risk of stroke and evaluate the association for per standard deviation (SD) reduction in BMD and incidence of stroke.

## Materials and methods

### Data source and search strategy

This systematic review and meta-analysis were conducted following the Preferred Reporting Items for Systematic Reviews and Meta-analyses (PRISMA) guidelines. It was prospectively registered in the International Prospective Register of Systematic Reviews. Two investigators independently searched MEDLINE, Embase, and the Cochrane Database of Systematic Reviews (CDSR) from the inception through January 2025. The search strategy was established using a combination of standardized terms including, but not limited to, ‘stroke’ or ‘intracranial hemorrhage’ or ‘cerebrovascular accident’ or ‘transient ischemic attack’ and ‘bone mineral density’ or ‘bone density’ or ‘bone mineral content.’ The detailed search strategy is presented in Supplementary methods. We then searched the reference lists of included articles and manually searched the reference lists of articles included in previous systematic reviews and meta-analyses to identify additional studies. No restrictions were imposed, and studies in all languages were eligible for inclusion, with translations performed as needed.

### Study eligibility and selection

Two authors independently assessed titles and abstracts to determine the eligibility of all studies identified in the literature search. Full-text articles were then assessed for inclusion. Discrepancies were resolved through consensus. Studies were included if they met the following criteria: (1) prospective or retrospective cohort designs; (2) the influencing factor of interest was BMD as a continuous variable or low BMD status as a categorical variable; (3) the outcome of interest was any type of stroke. Exclusion criteria included: (1) inability to extract outcomes of interest; (2) studies where participants had a history of stroke at baseline. This ensured that only studies assessing BMD as a predictor of new stroke events were included.

### Data extraction and quality assessment

Two of us extracted data from each eligible study independently and checked them, with discrepancies resolved via consensus. For each included study, the following details were collected: year of publication, geographical region where the study was conducted, study design, details and demographic characteristics of participants, BMD measure methods, duration of follow-up length, outcome measure, and details of outcomes reported. A low BMD level was defined as a T score < −1, consistent with the World Health Organization (WHO) criteria for osteopenia (10). To assess both quantitative (per SD reduction) and categorical (low BMD status) associations with stroke risk, we also accepted clinically diagnosed osteoporosis or osteoporotic fracture as proxies for low BMD status, as these conditions typically indicate T scores < −1 or lower. For studies using methods (e.g., broadband ultrasound attenuation, single-photon absorptiometry, or clinical diagnosis) other than dual-energy X-ray absorptiometry to measure BMD, we also accepted these results as proxies for BMD status, acknowledging that their equivalence to dual-energy X-ray absorptiometry varies.

The Newcastle-Ottawa Scale (NOS) was used to assess the methodological quality of the evidence and the risk of bias in the included studies (11). The total scores ranged from 0 (worst) to 9 (best) for cohort studies. A study is considered to have a low risk of bias if the total score is 7 or more. Any disagreements were resolved via discussion or, if needed, consultation with an external expert in biostatistics.

### Data synthesis and analysis

Statistical analyses were completed using RevMan software (version 5.4; Cochrane Collaboration). The relative risks (RRs) were used to assess the association of BMD with the incidence of stroke across studies. For each study, we included the RR adjusted for the highest number of covariates. We used the summary RRs if they were reported categorically. Hazard ratios (HRs) were substituted with RRs when reported, justified by the low stroke incidence during follow-up in the included studies, where HRs closely approximate RRs (12). We used the inverse variance (I-V) method to combine the effect sizes. We assessed statistical heterogeneity using the Q statistic and the I^2^ statistic. I^2^ values of more than 50% were considered significant heterogeneity. The random effect models were used to combine the effect sizes. Subgroup analyses were performed by sex and World Health Organization geographical region. A post-hoc subgroup analysis was performed by BMD measurement techniques. We did not evaluate funnel plot asymmetry to assess for publication bias because fewer than 10 studies were included in each comparison (13). The threshold for statistical significance was set at 0.05.

Given that the direct substitution of HRs using RRs may influence the accuracy of estimates, we performed a sensitivity analysis restricted to studies without substitution. To ensure robustness, a *post-hoc* sensitivity analysis by removing studies of high risk of bias (NOS score < 7) was also performed.

## Results

**Figure 1:**
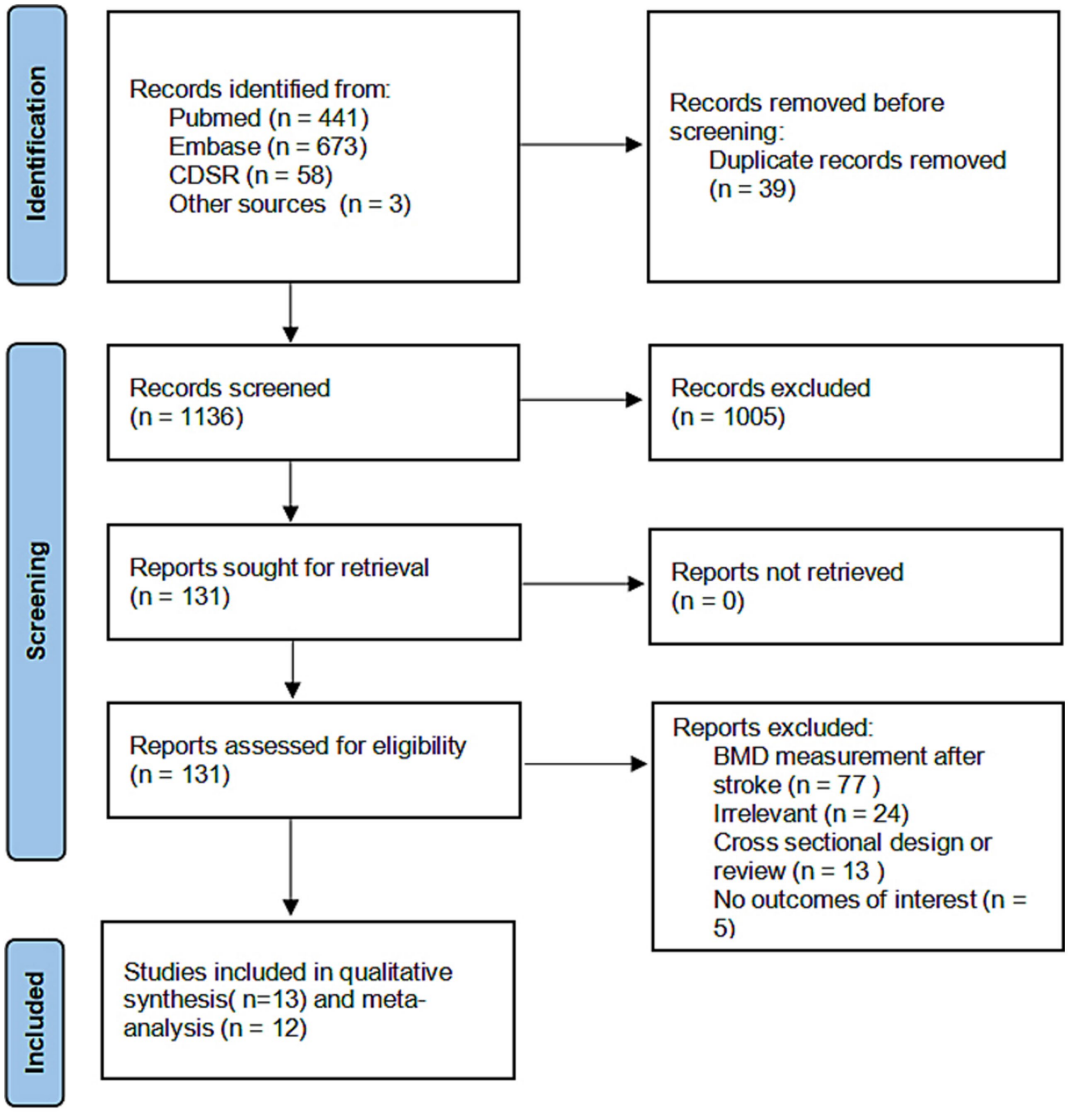
Flow diagram of studies selection.

The literature search identified 1,136 unique potentially relevant references. Three additional studies were obtained from a manual search of citations. One study was excluded because it was unclear whether the stroke in the study participants occurred during the follow-up period (14). After eligibility screening, 13 studies were included in the qualitative synthesis and 12 in the final meta-analysis (7, 8, 15–25). Study selection is illustrated in Figure 1.

### Study characteristics

In total, 146,758 individuals with 8,277 stroke cases were included in the 13 studies (7, 8, 15–25). There were 115,931 women, 25,123 men, and 5,704 persons with unreported sex. Of the included studies, six were from the Americas (15, 16, 19, 20, 22, 23), 3 from European (7, 8, 21), and 4 from the Western Pacific region (17, 18, 24, 25). There were 10 studies that reported outcomes with adjustment for at least three confounders (7, 8, 16–21, 23–25), consistently including age and sex, and variably adjusting for cardiovascular risk factors, lifestyle factors, and comorbidities (Table 1). In most studies (53.8%), measurements of BMD were conducted using single or dual-energy X-ray absorptiometry (8, 15, 16, 19–21, 23, 25). In another study, BMD was assessed using broadband ultrasound attenuation and velocity of sound (7). For the analysis of low BMD status and incident stroke, we also included two studies identifying low BMD by clinical diagnosis from medical records (18, 24) and one using a diagnosis of osteoporotic fracture (17), as these conditions typically reflect T scores < −1 or lower. Detailed information on the characteristics of the included studies is provided in Table 1.

**Table 1:** Characteristics of the included studies for data synthesis.

Source	Geographical region	Study design	Number of participants	Characteristic of participants	BMD measurement	Measure of effect	Adjustment
Bhatta (8)	European	Prospective cohort.	22,857	Adults from the second and third surveys of the HUNT Study, without history of cardiovascular diseases.	Single or dual-energy X-ray absorptiometry in the non-dominant distal forearm.	HR of stroke for each SD decrease in BMD.	Age, age-squared, BMI, physical activity, smoking status, alcohol use, and education level.
Browner (16)	Americas	Prospective cohort.	9,704	Women aged 65 years or older.	Single-photon absorptiometry in the distal radius, proximal radius, and calcaneus.	RR of stroke for each SD decrease in BMD.	Age, previous stroke, hypertension, diabetes mellitus, pack-years of cigarette smoking, and years of postmenopausal estrogen replacement.
Browner (15)	Americas	Prospective cohort.	4,024	Ambulatory women aged 65 years or older, without previous hip fractures or stroke.	Single-photon absorptiometry in the distal radius, proximal radius, and calcaneus.	HR of stroke for each SD decrease in BMD.	Age.
Chen (17)	Western Pacific	Population-based cohort.	4,175	Representative of general population, without previous stroke.	Low BMD: osteoporotic vertebral fracture.	HR of stroke in patients with osteoporosis.	Age, sex, comorbidities (including hypertension, diabetes, arrhythmia, and coronary heart disease), and exposure to medications (including aspirin, anticoagulants, lipid-lowering drugs and nitrates).
Lin (18)	Western Pacific	Population-based cohort.	15,480	Persons aged 50 years or older.	NR.	HR of stroke in patients with osteoporosis.	Sex, age, urbanization level, geographic region, hypertension, hyperlipidemia, diabetes, and hormone replacement therapy.
Mussolino (20)	Americas	Prospective cohort.	3,402	White and black persons aged 45 to 74 years.	Radiographic absorptiometry in the left hand.	RR of stroke for each SD decrease in BMD.	Age, smoking status, alcohol consumption, history of diabetes, history of heart disease, education, body mass index, recreational physical activity, and blood pressure medication.
Mussolino (19)	Americas	Prospective cohort.	5,272	White, black, and Mexican-American persons aged 50 years or older	Dual energy x-ray absorptiometry in the proximal femoral.	RR of stroke for each SD decrease in BMD.	Age at interview, gender, race-ethnicity, body mass index, calcium consumption, alcohol consumption, smoking status, physical activity, selfreported health status, hypertension, and history of diabetes.
Myint (7)	European	Prospective cohort.	14,290	Persons aged 40 to 79 years.	Broadband ultrasound attenuation and velocity of sound in bliteral calcaneum	RR of stroke for each SD decrease in BMD.	Age, sex, systolic blood pressure, cholesterol, smoking status, physical activity, body mass index, education level, social class, prevalent diabetes mellitus, prevalent myocardial infarction, use of lipid modifying and antihypertensives.
Nordström (21)	European	Prospective cohort.	4,302	Persons aged 40 to 75 years.	Dual-energy X-ray absorptiometry in femoral neck.	HR of stroke in patients with osteoporosis; HR of stroke for each SD decrease in BMD.	Age, sex, BMI, diabetes, current smoking and treatment for hypertension and hyperlipidemia.
O’Malley (22)	Americas	Retrospective cohort.	44,828	Women aged 55 to 89 years.	NR	HR of stroke in patients with osteoporosis.	None.
Szulc (23)	Americas	Prospective cohort.	743	Men aged 50 to 85 years.	Dual-energy X-ray absorptiometry in the lumbar spine, right hip, and whole Body.	NR.	Age, weight, height, smoking, occupational physical activity, education level, ischemic heart disease, hypertension, diabetes, 25(OH)D concentrations, and vitamin K–inhibiting anticoagulants and thiazides treatment.
Yu (24)	Western Pacific	Retrospective cohort.	12,535	Patients with end-stage renal disease undergoing incident dialysis.	NR	HR of stroke in patients with osteoporosis.	Age, sex, and comorbidities of diabetes, hypertension, hyperlipidemia, mental disorders, hepatitis B infection, and hepatitis C infection.
Zhou (25)	Western Pacific	Prospective cohort.	5,136	Postmenopausal women aged 50 years or older.	Dual energy X-ray absorptiometry in the femoral neck.	HR of stroke in patients with osteoporosis; HR of stroke for each SD decrease in BMD.	Age, BMI, current smokers, daily drinking, hypertension, diabetes mellitus, hypercholesterolemia.

### Quality assessment

The risk of bias assessment was conducted for each included study. Twelve studies (92.3%) were considered at low risk of bias and labeled as high quality (7, 8, 16–25). The primary source of bias was the “representativeness of the exposed cohort.” One study was considered at high risk of bias due to low “comparability” (15). The detailed quality assessment is presented in Supplementary Table 1.

### Per SD reduction in BMD and incident stroke

Nine studies with 69,731 participants and 3,272 stroke cases were included in the analysis of incident stroke for per SD reduction in BMD (7, 8, 15, 16, 19–21, 23, 25). Seven studies (7, 8, 15, 16, 21, 23, 25) reported that a reduction in BMD was associated with an increased risk of new-onset stroke during follow-up, with 6 of them at low risk of bias.

**Figure 2:**
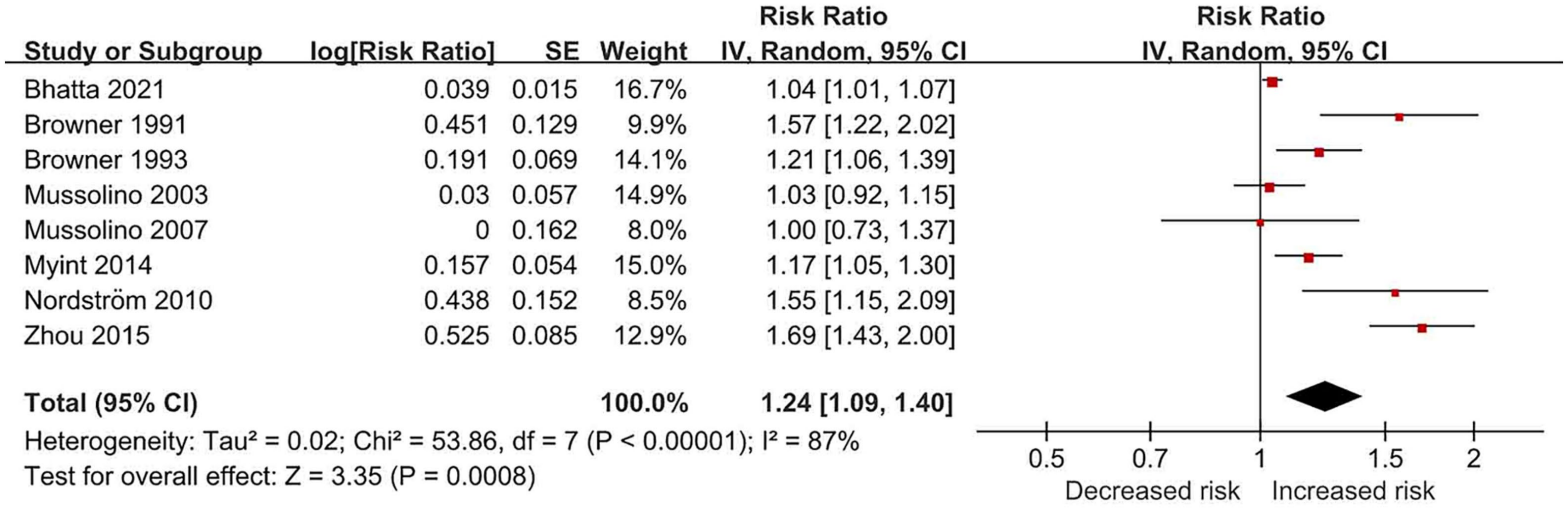
Forest plot of relative risk of incident stroke for per SD reduction in BMD.

Overall, a 1 SD reduction in BMD was associated with an increased risk of stroke (eight studies; RR, 1.24; 95% CI, 1.09–1.40; *p* < 0.001; I^2^ = 87%). The result is illustrated in Figure 2. Sensitivity analysis did not show significant differences (Supplementary Tables 2, 3). However, there was high statistical heterogeneity in these meta-analyses.

Subgroup analysis by sex (Supplementary Figure 1) showed a high level of heterogeneity which was mainly caused by studies of females. Per SD reduction in BMD was not associated with incident stroke in the male group (four studies; RR, 1.04; 95% CI, 0.99–1.10; *p* = 0.15; I^2^ = 0%), with a low level of heterogeneity. In the female group, the pooled RR increased without change in heterogeneity level (seven studies; RR, 1.28; 95% CI, 1.09–1.51; *p* = 0.002; I^2^ = 87%). In the mixed group (female + male), reduction in BMD was not associated with incident stroke (two studies; RR, 1.19; 95% CI, 0.72–1.97; *p* = 0.49; I^2^ = 84%). Subgroup analysis by geographical region (Supplementary Figure 2) showed that the study from the Western Pacific region mainly drove the increased risk (25). Post-hoc subgroup analysis by BMD measurement techniques (Supplementary Figure 3) showed that absorptiometry methods (dual-energy X-ray absorptiometry and single-photon absorptiometry) mainly drove the heterogeneity.

### Low BMD and incident stroke

Six studies (17, 18, 21, 22, 24, 25) with 86,456 participants and 5,292 stroke cases reported the association between osteoporosis (T score < −2.5) or osteopenia (T score < −1.0 and ≥ −2.5) and risk of incident stroke. Of the included six studies, five showed that low BMD was associated with an increased risk of incident stroke, with RR ranging from 1.13 (95%CI, 1.02–1.26) to 4.67 (95%CI, 2.13–10.23). All the included studies were at low risk of bias.

**Figure 3:**
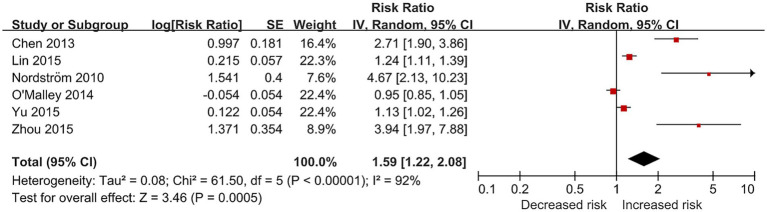
Forest plot of relative risk of incident stroke for osteoporosis or osteopenia.

Overall, low BMD status was associated with an increased risk of incident stroke, with pooled RR of 1.59 (six studies; 95% CI, 1.22–2.08; *p* < 0.001; I^2^ = 92%). The result is illustrated in Figure 3. Sensitivity analysis restricted to studies with results as HRs also showed an increased risk of incident stroke for low BMD status (Supplementary Table 2), with high statistical heterogeneity.

Subgroup analysis by sex (Supplementary Figure 4) reduced the level of heterogeneity and showed that low BMD was associated with an increased risk of incident stroke in the male group (two studies; RR, 1.27; 95% CI, 1.10–1.45; *p* < 0.001; I^2^ = 0%) and mixed group (two studies; RR, 3.16; 95% CI, 1.96–5.12; *p* < 0.001; I^2^ = 35%). While the association was not significant in the female group (four studies; RR, 1.20; 95% CI, 0.96–1.50; *p* = 0.11; I^2^ = 87%). Studies from the Western Pacific region showed an increased risk of stroke for individuals with osteoporosis or osteopenia (four studies; RR, 1.67; 95% CI, 1.22–2.29; *p* = 0.001; I^2^ = 91%), as well as study from European region (one study; RR, 4.67; 95%CI, 2.13–10.23; *p* < 0.001). Subgroup analysis by geographic region is illustrated in Supplementary Figure 5. Post-hoc subgroup analysis by BMD measurement techniques (Supplementary Figure 6) showed that studies without clear measurement techniques mainly drove the heterogeneity.

## Discussion

This systematic review and meta-analysis show that lower BMD is associated with a higher risk of incident stroke. The per SD reduction in BMD is associated with a 24% increase in the risk of stroke later in life. People with osteoporosis or osteopenia have a 59% increase in the risk of developing stroke. A 1 SD reduction in BMD is associated with a significantly increased risk of incident stroke (RR, 1.28; 95% CI, 1.09–1.51) among female persons but not among male persons (RR, 1.04; 95% CI, 0.99–1.10). Despite the sex and regional differences in effect sizes, the findings highlight the critical aspects of the relationship between bone health and stroke.

The relationship between BMD and incident stroke has been investigated for decades. As early as 1991, Browner et al. found a 70% increase in stroke mortality for each SD decrease in BMD at the proximal radius in a prospective cohort of more than 9,000 persons (16). Two years later, Browner et al. investigated the association between low bone density and the risk of stroke in 4,024 women aged 65 years or older (15). The risk of developing stroke was increased by 31% for each SD decrease in BMD at the calcaneus. However, the authors do not believe that low BMD causes stroke directly. They suggested that there could be factors related to the integrity of the intracerebral vascular that link BMD with stroke. Notably, although the authors were aware that potential factors could confound the association, they did not adjust possible confounders when reporting the results. In recent years, the possible mechanisms are still being investigated (26, 27).

In 2014, a prospective cohort and meta-analysis study by Myint et al. reported that low BMD was an independent factor for the risk of incident stroke in the middle and older general population. The meta-analysis section included three additional studies and found an inverse relationship between BMD and risk of incident stroke (RR, 1.12; 95%CI, 1.04–1.22) (7). However, the number of included studies was small. A meta-analysis by Qu et al. reported that low BMD was not associated with the risk of stroke mortality (HR 1.08; 95% CI, 0.89–1.28) (9). The small number of studies and participants also limited the strength of conclusions. Although our conclusions are similar to previous studies, the strength of our results and new findings are significant. Although one study was assessed as having a high risk of bias due to limited comparability, its inclusion did not substantially alter the meta-analysis results (15).

During literature screening, we noticed that many studies focused on the risk of osteoporosis or reduction in BMD in patients with stroke. Patients experience accelerated bone loss after stroke, most pronounced in the paretic limbs (28). Jorgensen et al. observed an up to 24% loss of BMD in the paretic proximal humerus in patients 1 year after stroke (29). The loss of BMD in the acute post-stroke period is associated with poor functional outcomes (30). Immobilization resulting from a stroke can lead to decreased mechanical loading on the bones, further resulting in bone loss. These can contribute to increased fracture risk and mortality in stroke patients (31). A multi-center study of 23,751 stroke patients and 23,751 age−/sex-matched controls showed that the 2-year rate of low-trauma fractures was 5.7% in those with stroke, significantly higher than the controls (adjusted HR, 1.47; 95% CI 1.35–1.60) (32). From above, the condition of stroke and low BMD are aggravated by each other. All the studies included in the meta-analysis excluded patients with stroke at baseline or precluded them in risk assessment, which is essential to eliminate the confounding of the previous stroke.

Growing evidence suggests that low BMD or osteoporosis is associated with an increased risk of stroke. However, the underlying mechanisms are not fully understood. Several possibilities have been proposed to explain this association, including shared risk factors, common pathophysiological pathways, and genetic factors (7, 28, 33, 34). Low BMD and osteoporosis share several risk factors with stroke, including age, female gender, physical inactivity, and low estrogen level (35, 36). These factors have been reported to contribute to the development of osteoporosis and stroke, which may partially explain the observed association between low BMD and stroke. Several genetic variants have been identified to increase the risk of both BMD and stroke, suggesting a potential genetic overlap between the two conditions. The vitamin D receptor (VDR) gene plays a critical role in bone metabolism and has also been linked to stroke risk (37, 38). In addition, the estrogen receptor alpha (ESR1) gene is involved in the regulation of bone metabolism and has also been linked to stroke risk (39, 40). The reported polymorphisms in the ESR1 gene included the PvuII polymorphism and the XbaI polymorphism. Another study by Frost et al. implies that alterations in osteoprotegerin-mediated signaling in the vascular may be involved in the pathophysiology of hemorrhagic stroke, further supporting a shared genetic basis for these conditions (33).

The remaining possibilities underlying the association may be due to chance or bias. However, given the statistical significance of the results in most studies included and the significance of low BMD in cardiovascular disease, the chance is an unlikely explanation. There may be selection bias and publication bias in the present study. Given that the test power of publication bias assessment is usually too low in the meta-analysis with fewer than 10 studies, we did not evaluate funnel plot asymmetry to assess for publication bias.

The observed association between reduction in BMD and stroke has important management implications. First, stroke patients should be evaluated for osteoporosis, particularly those with risk factors such as female gender, advanced age, and prolonged immobilization (41). BMD testing and appropriate interventions, such as lifestyle modifications, pharmacologic therapy, and fall prevention measures, should be considered to reduce this population’s risk of osteoporotic fractures. Second, osteoporosis patients should be evaluated for stroke risk factors and managed accordingly, including control of hypertension, diabetes, and dyslipidemia, as well as lifestyle modifications to reduce the risk of stroke or stroke recurrence (42, 43). Third, future studies should investigate the mechanisms underlying the association and evaluate the effectiveness of treatment against bone loss in reducing the incidence of stroke.

The stronger link between reduced BMD and stroke risk in females versus males suggests sex-specific mechanisms. In females, postmenopausal estrogen decline accelerates BMD loss (44). This decline also reduces endothelial protection and increases inflammation which are associated with the risk of stroke (45). Estrogen supports bone density and vascular health by boosting nitric oxide and lowering oxidative stress (46). The effects lost after menopause, heightening stroke risk as BMD drops. Males maintain higher BMD and testosterone that may protect vascular integrity and lessen stroke risk despite BMD reduction (47). In addition, females’ longer lifespan also extends their exposure to low BMD, worsening vascular damage over time. Future studies could test if estrogen loss drives this link in females or if prolonged low BMD amplifies their stroke risk.

The rationale for conducting subgroup analyses by sex and geographical region stems from their established roles in BMD and stroke epidemiology. Women experience accelerated BMD decline post-menopause due to estrogen loss, a known risk factor for both osteoporosis and cardiovascular events, including stroke (25, 36). This aligns with our findings of a stronger association in females compared to males for per SD BMD reduction. Geographical region was chosen to explore regional variations in stroke incidence and BMD-related factors, such as dietary calcium intake, physical activity, and genetic predispositions (48).

The high statistical heterogeneity observed in our meta-analysis likely stems from several sources, as evidenced by subgroup analysis. For the low BMD status analysis, studies lacking clear measurement technique descriptions were the main source of heterogeneity, suggesting potential inconsistencies in diagnostic criteria or unreported methods. Participant demographics also played a role. For the per SD reduction in BMD analysis, the female subgroup exhibited persistently high heterogeneity, while male and mixed groups showed lower variability. Although meta-regression could quantify these contributions, the small number of studies per comparison prevented its application. In addition, the current study included studies using clinical diagnoses of osteoporosis or osteoporotic fracture as proxies for low BMD status (T score < −1), as these conditions indicate significant bone loss. Variability in BMD assessment methods may contribute to high heterogeneity and affect effect sizes. Specifically, dual-energy X-ray absorptiometry offers higher precision for central skeletal sites, while single-photon absorptiometry and ultrasound measure peripheral sites with varying sensitivity, potentially underestimating or overestimating stroke risk associations (49). These findings underscore the need for standardized BMD assessment and detailed reporting in future research to reduce heterogeneity and enhance comparability.

This systematic review and meta-analysis have several limitations. First, there was significant methodological heterogeneity among selected studies in terms of measurement of BMD, characteristics of participants, and confirmation of stroke. Despite subgroup analyses, most results with positive findings were accompanied by high statistical heterogeneity. Second, the measurement of BMD was conducted at baseline (the beginning of each study).No studies checked the BMD levels during follow-up. Without serial measurements, studies may not fully capture dynamic changes in bone health influencing stroke incidence, which may overestimate the real impact of BMD on incident stroke. Future studies should incorporate longitudinal BMD monitoring to better assess its temporal relationship with stroke risk. Third, although 10 studies adjusted for key confounders such as age, sex, and cardiovascular risk factor, residual confounding from unmeasured genetic factors (e.g., VDR and ESR1 polymorphisms) and laboratory indicators (e.g., vitamin D levels, inflammatory markers) may persist. These unadjusted variables linked to both bone metabolism and stroke risk, potentially influencing the BMD-stroke association (35, 38). Fourth, we did not formally test for publication bias due to limited studies per comparison. This raises the possibility that small-study effects or selective reporting could bias our pooled estimates. Additionally, BMD measurement methods varied across studies, with most using dual-energy X-ray absorptiometry, while others employed broadband ultrasound, single-photon absorptiometry, clinical diagnosis, or not reported. Although ultrasound has shown a meaningful correlation with DXA in assessing bone density (50), and clinical diagnoses reflect real-world practice, these differences may introduce heterogeneity and affect comparability, potentially overestimating or underestimating the true association with stroke risk.

## Conclusion

Reduction in BMD is associated with a significantly increased risk of developing stroke. Female persons have a higher risk than male persons. People with osteoporosis or osteopenia have an increased risk of incident stroke. The findings highlight the critical relationship between bone health and stroke.

## Data Availability

The raw data supporting the conclusions of this article will be made available by the authors, without undue reservation.

## References

[1] Feigin VL Nguyen G Cercy K Johnson CO Alam T Parmar PG. Global, regional, and country-specific lifetime risks of stroke, 1990 and 2016. N Engl J Med. (2018) 379:2429–37. doi: 10.1056/NEJMoa1804492, PMID: 30575491 PMC6247346

[2] Katan M Luft A. Global burden of stroke. Semin Neurol. (2018) 38:208–11. doi: 10.1055/s-0038-1649503, PMID: 29791947

[3] Ma Q Li R Wang L Yin P Wang Y Yan C. Temporal trend and attributable risk factors of stroke burden in China, 1990-2019: an analysis for the global burden of disease study 2019. Lancet Public Health. (2021) 6:e897–906. doi: 10.1016/S2468-2667(21)00228-0, PMID: 34838196 PMC9047702

[4] Virani SS Alonso A Aparicio HJ Benjamin EJ Bittencourt MS Callaway CW. Heart disease and stroke statistics-2021 update: a report from the american heart association. Circulation. (2021) 143:e254–743. doi: 10.1161/CIR.0000000000000950, PMID: 33501848 PMC13036842

[5] Saleh I Akbar A Hasan HS Yulisa ND Aprilya D. Clinical characteristics and bone mineral density score in Post-stroke neuromuscular deficit. J Clin Med Res. (2025) 17:119–24. doi: 10.14740/jocmr6070, PMID: 39981337 PMC11835557

[6] Lam FM Bui M Yang FZ Pang MY. Chronic effects of stroke on hip bone density and tibial morphology: a longitudinal study. Osteoporos Int. (2016) 27:591–603. doi: 10.1007/s00198-015-3307-7, PMID: 26329101

[7] Myint PK Clark AB Kwok CS Loke YK Yeong JK Luben RN. Bone mineral density and incidence of stroke: european prospective investigation into cancer-Norfolk population-based study, systematic review, and meta-analysis. Stroke. (2014) 45:373–82. doi: 10.1161/STROKEAHA.113.002999, PMID: 24399373

[8] Bhatta L Cepelis A Vikjord SA Malmo V Laugsand LE Dalen H. Bone mineral density and risk of cardiovascular disease in men and women: the hunt study. Eur J Epidemiol. (2021) 36:1169–77. doi: 10.1007/s10654-021-00803-y, PMID: 34515906 PMC8629874

[9] Qu X Huang X Jin F Wang H Hao Y Tang T. Bone mineral density and all-cause, cardiovascular and stroke mortality: a meta-analysis of prospective cohort studies. Int J Cardiol. (2013) 166:385–93. doi: 10.1016/j.ijcard.2011.10.114, PMID: 22112679

[10] Kanis JA. Assessment of fracture risk and its application to screening for postmenopausal osteoporosis: synopsis of a WHO report. Osteoporos Int. (1994) 4:368–81. doi: 10.1007/BF01622200, PMID: 7696835

[11] GA Wells BSDO. The newcastle-ottawa scale (nos) for assessing the quality of nonrandomised studies in meta-analyses. (2021) Available online at: https://www.ohri.ca/programs/clinical_epidemiology/oxford.asp. (Accessed January 15, 2025).

[12] Tierney JF Stewart LA Ghersi D Burdett S Sydes MR. Practical methods for incorporating summary time-to-event data into meta-analysis. Trials. (2007) 8:16. doi: 10.1186/1745-6215-8-16, PMID: 17555582 PMC1920534

[13] Sterne JA Sutton AJ Ioannidis JP Terrin N Jones DR Lau J. Recommendations for examining and interpreting funnel plot asymmetry in meta-analyses of randomised controlled trials. BMJ. (2011) 343:d4002. doi: 10.1136/bmj.d4002, PMID: 21784880

[14] Zhu B Yang J Zhou Z Ling X Cheng N Wang Z. Total bone mineral density is inversely associated with stroke: a family osteoporosis cohort study in rural China. QJM. (2022) 115:228–34. doi: 10.1093/qjmed/hcaa339, PMID: 33453113

[15] Browner WS Pressman AR Nevitt MC Cauley JA Cummings SR. Association between low bone density and stroke in elderly women. The study of osteoporotic fractures. Stroke. (1993) 24:940–6. doi: 10.1161/01.str.24.7.940, PMID: 8322393

[16] Browner WS Seeley DG Vogt TM Cummings SR. Non-trauma mortality in elderly women with low bone mineral density. Study of osteoporotic fractures research group. Lancet. (1991) 338:355–8. doi: 10.1016/0140-6736(91)90489-c, PMID: 1677708

[17] Chen YC Wu JC Liu L Huang WC Cheng H Chen TJ. Hospitalized osteoporotic vertebral fracture increases the risk of stroke: a population-based cohort study. J Bone Miner Res. (2013) 28:516–23. doi: 10.1002/jbmr.1722, PMID: 22836881

[18] Lin CH Chang WC Kuo CN Yu HC Yang CC Lin YW. A population-based five-year study on the risk of stroke in patients with osteoporosis in Taiwan. Bone. (2015) 72:9–13. doi: 10.1016/j.bone.2014.11.007, PMID: 25460575

[19] Mussolino ME Armenian HK. Low bone mineral density, coronary heart disease, and stroke mortality in men and women: the third national health and nutrition examination survey. Ann Epidemiol. (2007) 17:841–6. doi: 10.1016/j.annepidem.2007.06.005, PMID: 17728148

[20] Mussolino ME Madans JH Gillum RF. Bone mineral density and stroke. Stroke. (2003) 34:e20–2. doi: 10.1161/01.STR.0000065826.23815.A5, PMID: 12663880

[21] Nordström A Eriksson M Stegmayr B Gustafson Y Nordström P. Low bone mineral density is an independent risk factor for stroke and death. Cerebrovasc Dis. (2010) 29:130–6. doi: 10.1159/000262308, PMID: 19955736

[22] O'Malley CD Tran N Zapalowski C Daizadeh N Olenginski TP Cauley JA. Multimorbidity in women with and without osteoporosis: results from a large us retrospective cohort study 2004-2009. Osteoporos Int. (2014) 25:2117–30. doi: 10.1007/s00198-014-2740-3, PMID: 24859882

[23] Szulc P Samelson EJ Kiel DP Delmas PD. Increased bone resorption is associated with increased risk of cardiovascular events in men: the minos study. J Bone Miner Res. (2009) 24:2023–31. doi: 10.1359/jbmr.090531, PMID: 19453264 PMC2791516

[24] Yu TM Lin CL Shu KH Liu YL Chen CH Huang ST. Increased risk of cardiovascular events in end-stage renal disease patients with osteoporosis: a nationwide population-based cohort study. Osteoporos Int. (2015) 26:785–93. doi: 10.1007/s00198-014-2982-0, PMID: 25491767

[25] Zhou R Liu D Li R Zhou S Cui M Chen L. Low bone mass is associated with stroke in chinese postmenopausal women: the Chongqing osteoporosis study. Cell Biochem Biophys. (2015) 71:1695–701. doi: 10.1007/s12013-014-0392-8, PMID: 25481304

[26] Zheng L Liu M Gao Y Liu D Tian J. The association between total body bone mineral density and stroke: a Mendelian randomization analyses. QJM (2023) 116:471–72. doi: 10.1093/qjmed/hcac225 36194020

[27] Tsai YL Chuang YC Cheng YY Deng YL Lin SY Hsu CS. Low Bone Mineral Density as a Predictor of Mortality and Infections in Stroke Patients: A Hospital-Based Study. J Clin Endocrinol Metab (2024) 109:3055–64. doi: 10.1210/clinem/dgae365 38795366

[28] Yoo SD Kim TW Oh BM Lee SA Kim C Chung HY. Discordance between spine-hip and paretic-nonparetic hip bone mineral density in hemiplegic stroke patients: a multicenter retrospective study. Ann Rehabil Med. (2024) 48:413–22. doi: 10.5535/arm.240079, PMID: 39736498 PMC11703605

[29] Jorgensen L Jacobsen BK. Changes in muscle mass, fat mass, and bone mineral content in the legs after stroke: a 1 year prospective study. Bone. (2001) 28:655–9. doi: 10.1016/s8756-3282(01)00434-3, PMID: 11425655

[30] Lee SB Cho AH Butcher KS Kim TW Ryu SY Kim YI. Low bone mineral density is associated with poor clinical outcome in acute ischemic stroke. Int J Stroke. (2013) 8:68–72. doi: 10.1111/j.1747-4949.2011.00714.x, PMID: 22151871

[31] Lee HY Park JH Lee H Kim TW Yoo SD. Does hip bone density differ between paretic and non-paretic sides in hemiplegic stroke patients? And its relationship with physical impairment. J Bone Metab. (2020) 27:237–46. doi: 10.11005/jbm.2020.27.4.237, PMID: 33317227 PMC7746477

[32] Kapral MK Fang J Alibhai SM Cram P Cheung AM Casaubon LK. Risk of fractures after stroke: results from the Ontario stroke registry. Neurology. (2017) 88:57–64. doi: 10.1212/WNL.0000000000003457, PMID: 27881629 PMC5200858

[33] Frost ML Grella R Millasseau SC Jiang BY Hampson G Fogelman I. Relationship of calcification of atherosclerotic plaque and arterial stiffness to bone mineral density and osteoprotegerin in postmenopausal women referred for osteoporosis screening. Calcif Tissue Int. (2008) 83:112–20. doi: 10.1007/s00223-008-9153-2, PMID: 18612580

[34] Smith C Sim M Ilyas Z Gilani SZ Suter D Reid S. Automated abdominal aortic calcification and major adverse cardiovascular events in people undergoing osteoporosis screening: the Manitoba bone mineral density registry. J Bone Miner Res. (2025) 40:323–31. doi: 10.1093/jbmr/zjae208, PMID: 39749990 PMC11909729

[35] Rozenberg S Al-Daghri N Aubertin-Leheudre M Brandi ML Cano A Collins P. Is there a role for menopausal hormone therapy in the management of postmenopausal osteoporosis? Osteoporos Int. (2020) 31:2271–86. doi: 10.1007/s00198-020-05497-8, PMID: 32642851 PMC7661391

[36] Tamaki J Iki M Hirano Y Sato Y Kajita E Kagamimori S. Low bone mass is associated with carotid atherosclerosis in postmenopausal women: the japanese population-based osteoporosis (jpos) cohort study. Osteoporos Int. (2009) 20:53–60. doi: 10.1007/s00198-008-0633-z, PMID: 18496639

[37] Pirrotta F Cavati G Mingiano C Merlotti D Nuti R Gennari L. Vitamin D deficiency and cardiovascular mortality: retrospective analysis "Siena osteoporosis" cohort. Nutrients. (2023) 15:3303. doi: 10.3390/nu15153303, PMID: 37571241 PMC10421091

[38] Foroughinia F Morovati N Safari A Dianatpour M Jamhiri I Heydari ST. Association between fok1 and taqi polymorphisms of vitamin d receptor gene with the severity of stenosis and calcification in carotid bulb in patients with ischemic stroke. J Clin Neurosci. (2022) 97:115–20. doi: 10.1016/j.jocn.2022.01.009, PMID: 35091316

[39] Lazaros L Markoula S Xita N Giannopoulos S Gogou P Lagos G. Association of estrogen receptor-alpha gene polymorphisms with stroke risk in patients with metabolic syndrome. Acta Neurol Scand. (2008) 117:186–90. doi: 10.1111/j.1600-0404.2007.00926.x, PMID: 17854418

[40] Wang CL Tang XY Chen WQ Su YX Zhang CX Chen YM. Association of estrogen receptor alpha gene polymorphisms with bone mineral density in chinese women: a meta-analysis. Osteoporos Int. (2007) 18:295–305. doi: 10.1007/s00198-006-0239-2, PMID: 17089081

[41] Knight J Wu J Pike K. Screening for osteoporosis following stroke: a systematic review and Meta-analysis. Osteoporos Int. (2024) 35:1615–23. doi: 10.1016/S1474-4422(18)30500-3, PMID: 38922398 PMC11364682

[42] Wu S Wu B Liu M. Stroke in China: advances and challenges in epidemiology, prevention, and management. Lancet Neurol. (2021) 20:394–405. doi: 10.1016/S1474-4422(21)00040-4 30878104

[43] Oge DD Arsava EM Topcuoglu MA. Impact of low muscle mass and bone mineral density on long-term outcomes of acute ischemic stroke: A prospective study. Clin Nutr ESPEN (2025) 66:69–75. doi: 10.1016/j.clnesp.2024.12.021 39743138

[44] Devine A Dick IM Dhaliwal SS Naheed R Beilby J Prince RL. Prediction of incident osteoporotic fractures in elderly women using the free estradiol index. Osteoporos Int (2005) 16:216–221. doi: 10.1007/s00198-004-1674-6 15197544

[45] Li X Zhang W Zhang W. Inflammation and aging: signaling pathways and intervention therapies. Signal Transduct Target Ther. (2023) 8:239. doi: 10.1038/s41392-023-01502-8, PMID: 37291105 PMC10248351

[46] Zhao D Guallar E Ouyang P Subramanya V Vaidya D Ndumele CE. Endogenous sex hormones and incident cardiovascular disease in post-menopausal women. J Am Coll Cardiol. (2018) 71:2555–66. doi: 10.1016/j.jacc.2018.03.530, PMID: 29852978 PMC5986086

[47] Mohamad NV Soelaiman IN Chin KY. A concise review of testosterone and bone health. Clin Interv Aging. (2016) 11:1317–24. doi: 10.2147/CIA.S115253, PMID: 27703340 PMC5036835

[48] Cheng L Wang S Tang H. Regional differences in bone mineral density and stroke risk: a population-based cohort study. Osteoporos Int. (2024) 35:1289–98. doi: 10.1007/s00198-024-07123-3, PMID: 38760503

[49] Hans D Dargent-Molina P Schott AM Sebert JL Cormier C Kotzki PO. Ultrasonographic heel measurements to predict hip fracture risk in the elderly: the EPIDOS prospective study. Lancet. (1996) 348:511–4. doi: 10.1016/s0140-6736(95)11456-4, PMID: 8757153

[50] Glüer CC Wu CY Genant HK. Broadband ultrasound attenuation signals depend on trabecular orientation: an in vitro study. Osteoporos Int. (1993) 3:185–91. doi: 10.1007/BF01623674 8338973

