# Supplementary material for: Association between bone mineral density and stroke: a meta-analysis
Source: Front Neurol. 2025 Mar 26;16:1561559. doi: 10.3389/fneur.2025.1561559 (PMC11978661; doi:10.3389/fneur.2025.1561559)
Supplement: Supplementary file 1 [file Table_1.docx]

**Supplementary material**

**Methods. Search Strategy**

**Medline via Pubmed**

#1 "stroke" OR "brain ischemia" OR "hypoxia ischemia, brain" OR "Cerebral Infarction" OR "intracranial hemorrhages"/MeSH Terms 308,647

#2 "stroke" OR "brain isch*" OR "brain hypoxia ischemia" OR "Cerebral Infarction" OR "intracranial hemorrhag*" OR "brain infarct*" OR "transient ischaemic attack" OR "transient ischemic attack" OR "intracranial hemorrhage" OR "intracranial hemorrhage" OR "intracranial haemorrhage" OR "intracranial haemorrhage" OR "cerebrovascular accident" OR "brain vascular accident" OR "cerebral infarct*" OR "cerebrovascular occlusion"/Title/Abstract 381,224

#3 #1 OR #2 509,616

#4 "Bone Density"/MeSH Terms 63,786

#5 "bone mineral densit*" OR "density of bone" OR "bone densit*" OR "bone strength*" OR "bone mineral content*" OR "bone loss" OR "osseous densit*"/Title/Abstract 103,925

#6 #4 OR #5 120,085

#7 #3 AND #6 600

#8 "review"/Publication Type 3,442,629

#9 #7 NOT #8 441

**Embase**

#1 'cerebrovascular accident'/exp 480,549

#2 'brain ischemia'/exp 228,213

#3 'brain infarction'/exp 97,548

#4 'brain hemorrhage'/exp 201,517

#5 'stroke patient'/exp 50,093

#6 #1 OR #2 OR #3 OR #4 OR #5 813,926

#7 'stroke' OR 'brain isch*' OR 'brain hypoxia ischemia' OR 'cerebral infarction' OR 'intracranial hemorrhag*' OR 'brain infarct*' OR 'transient ischaemic attack' OR 'transient ischemic attack' OR 'intracranial hemorrhage' OR 'intracranial haemorrhage' OR 'cerebrovascular accident' OR 'brain vascular accident' OR 'cerebral infarct*' OR 'cerebrovascular occlusion':ab,kw,ti 871,731

#8 #6 OR #7 994,393

#9 'bone density'/exp 123,829

#10 'bone mineral densit*' OR 'density of bone' OR 'bone densit*' OR 'bone strength*' OR 'bone mineral content*' OR 'bone loss' OR 'osseous densit*':ab,kw,ti 196,961

#11 #9 OR #10 197,359

#12 #8 AND #11 2,398

#13 #8 AND #11 AND [animals]/lim 134

#14 #12 NOT #13 2,264

#15 #12 NOT #13 AND ([conference review]/lim OR [editorial]/lim OR [erratum]/lim OR [review]/lim OR [preprint]/lim) 687

#16 #14 NOT #15 1,577

#17 #14 NOT #15 AND ([medline]/lim OR [pubmed-not-medline]/lim) 904

#18 #16 NOT #17 673

**Cochrane laboratory**

#1 MeSH descriptor: [Stroke] explode all trees 18,294

#2 MeSH descriptor: [Brain Ischemia] explode all trees 6,052

#3 MeSH descriptor: [Hypoxia-Ischemia, Brain] explode all trees 378

#4 MeSH descriptor: [Cerebral Infarction] explode all trees 1,728

#5 MeSH descriptor: [Intracranial Hemorrhages] explode all trees 3,281

#6 #1 or #2 or #3 or #4 or #5 22,032

#7 ('stroke' OR 'brain isch*' OR 'brain hypoxia ischemia' OR 'Cerebral Infarction' OR 'intracranial hemorrhag*' OR 'brain infarct*' OR 'transient ischaemic attack' OR 'transient ischemic attack' OR 'intracranial hemorrhage' OR 'intracranial hemorrhage' OR 'intracranial haemorrhage' OR 'intracranial haemorrhage' OR 'cerebrovascular accident' OR 'brain vascular accident' OR 'cerebral infarct*' OR 'cerebrovascular occlusion'):ti,ab,kw 77305

#8 MeSH descriptor: [Bone Density] explode all trees 6,108

#9 ('bone mineral densit*' OR 'density of bone' OR 'bone densit*' OR 'bone strength*' OR 'bone mineral content*' OR 'bone loss' OR 'osseous densit*'):ti,ab,kw 87,975

#10 #8 or #9 89,092

#11 #6 and #10 58

**Table 1. Quality assessment of included cohort studies via Newcastle-Ottawa Scale.**

| Source | Selection | | | | Comparability | Outcome | | | Total |
| --- | --- | --- | --- | --- | --- | --- | --- | --- | --- |
|  | Representativeness of the exposed cohort | Selection of the non-exposed cohort | Ascertainment of exposure | Demonstration that outcome of interest was not present at the start of the study | Comparability of cohorts on the basis of the design or analysis | Assessment of outcome | Was follow-up long enough for outcomes to occur | Adequacy of the follow-up of cohorts |  |
| Bhatta 2021 | 1 | 1 | 1 | 1 | A (1)  B (1) | 1 | 1 | 1 | 9 |
| Browner 1991 | 0 | 1 | 1 | 0 | A (1)  B (1) | 1 | 1 | 1 | 7 |
| Browner 1993 | 0 | 1 | 1 | 1 | A (0)  B (0) | 1 | 1 | 1 | 6 |
| Chen 2013 | 0 | 1 | 1 | 1 | A (1)  B (1) | 1 | 1 | 1 | 8 |
| Lin 2015 | 1 | 1 | 1 | 1 | A (1)  B (1) | 1 | 1 | 1 | 9 |
| Mussolino 2003 | 1 | 1 | 1 | 1 | A (1)  B (1) | 1 | 1 | 1 | 9 |
| Mussolino 2007 | 0 | 1 | 1 | 1 | A (1)  B (1) | 1 | 1 | 1 | 8 |
| Myint 2014 | 1 | 1 | 1 | 1 | A (1)  B (1) | 1 | 1 | 1 | 9 |
| Nordström 2010 | 1 | 1 | 1 | 1 | A (1)  B (1) | 1 | 1 | 1 | 9 |
| O'Malley 2014 | 1 | 1 | 1 | 1 | A (0)  B (0) | 1 | 1 | 1 | 7 |
| Szulc 2009 | 1 | 1 | 1 | 0 | A (1)  B (1) | 0 | 1 | 1 | 7 |
| Yu 2015 | 1 | 1 | 1 | 1 | A (1)  B (1) | 0 | 1 | 1 | 8 |
| Zhou 2015 | 1 | 1 | 1 | 1 | A (1)  B (1) | 1 | 1 | 1 | 9 |

For comparability: A, adjusted for at least 3 confounders; B, adjusted for at least one classical cerebrovascular risk factor.

**Table 2.** Sensitivity analysis restricted to studies reported results as HRs**.**

|  | **Studies** | **Participants** | **Pooled HR** | **95%CI** | ***P* value** | **I2** |
| --- | --- | --- | --- | --- | --- | --- |
| Per SD reduction in BMD and incident stroke | 4 | 36,319 | 1.32 | 1.04 - 1.68 | 0.02 | 93% |
| Low BMD and incident stroke | 4 | 41,628 | 1.91 | 1.37 - 2.66 | < 0.001 | 93% |

**Table 3.** Sensitivity analysis restricted to studies of low risk of bias**.**

|  | **Studies** | **Participants** | **Pooled RR** | **95%CI** | ***P* value** | **I2** |
| --- | --- | --- | --- | --- | --- | --- |
| Per SD reduction in BMD and incident stroke | 7 | 65,707 | 1.24 | 1.08 - 1.43 | < 0.001 | 88% |
| Low BMD and incident stroke | 6 | 86,456 | 1.59 | 1.22-2.08 | < 0.001 | 92% |

**

**

**Figure 1.** Forest plot of relative risk of incident stroke for per SD reduction in BMD by sex.





**Figure 2.** Forest plot of relative risk of incident stroke for per SD reduction in BMD by geographic region.

**
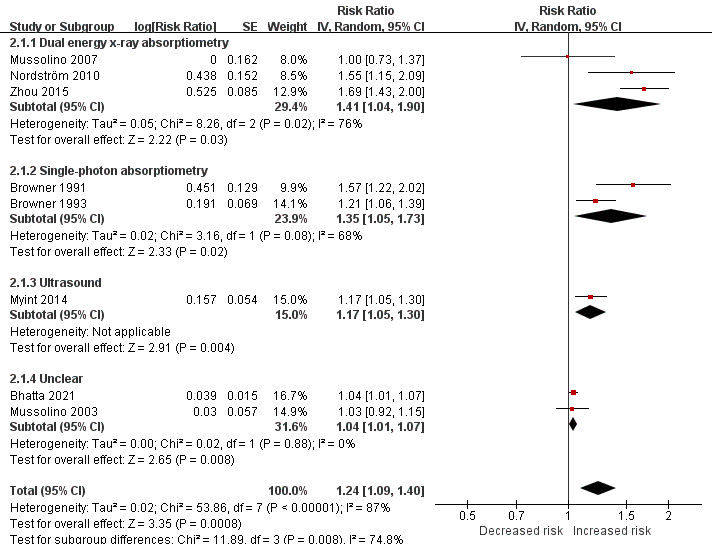
**

**Figure 3.** Forest plot of relative risk of incident stroke for per SD reduction in BMD by measurement techniques.





**Figure 4.** Forest plot of relative risk of incident stroke for low BMD by sex.

**

**

**Figure 5.** Forest plot of relative risk of incident stroke for low BMD by geographic region.

**
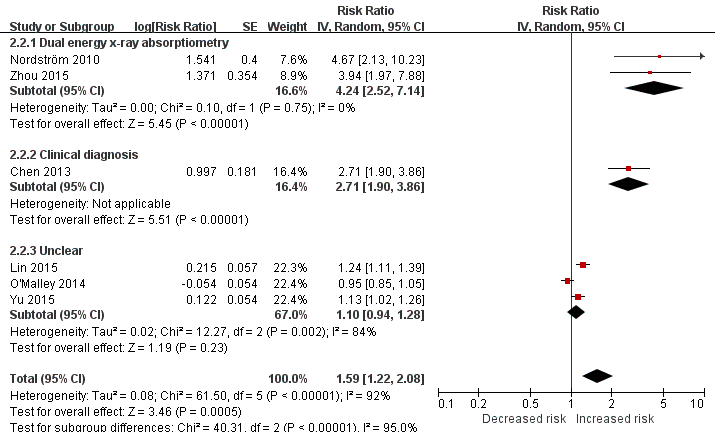

Figure 6.** Forest plot of relative risk of incident stroke for low BMD by measurement techniques.
